# Amyloid-β precursor protein promotes cell proliferation and motility of advanced breast cancer

**DOI:** 10.1186/1471-2407-14-928

**Published:** 2014-12-10

**Authors:** Seunghwan Lim, Byoung Kwon Yoo, Hae-Suk Kim, Hannah L Gilmore, Yonghun Lee, Hyun-pil Lee, Seong-Jin Kim, John Letterio, Hyoung-gon Lee

**Affiliations:** Department of Pediatrics, Case Comprehensive Cancer Center, Case Western Reserve University School of Medicine, 2103 Cornell Road, Cleveland, OH 44106 USA; Department Pathology, Case Western Reserve University School of Medicine, 2103 Cornell Road, Cleveland, OH 44106 USA; Department Pharmacology, Case Western Reserve University School of Medicine, Cleveland, OH 44106 USA; Department of Human and Molecular Genetics, Virginia Commonwealth University School of Medicine, Richmond, VA USA; CHA Cancer Institute, CHA University, Seoul, 135-081 Korea; University Hospitals Rainbow Children’s Hospital, The Angie Fowler Adolescent and Young Adult Cancer Institute, Cleveland, OH 44106 USA

**Keywords:** AKT, Amyloid-β precursor protein, Apoptosis, Breast cancer, Invasion, p27^kip1^

## Abstract

**Background:**

Amyloid-β precursor protein (APP) is a highly conserved single transmembrane protein that has been linked to Alzheimer disease. Recently, the increased expression of APP in multiple types of cancers has been reported where it has significant correlation with the cancer cell proliferation. However, the function of APP in the pathogenesis of breast cancer has not previously been determined. In this study, we studied the pathological role of APP in breast cancer and revealed its potential mechanism.

**Methods:**

The expression level of APP in multiple breast cancer cell lines was measured by Western blot analysis and the breast cancer tissue microarray was utilized to analyze the expression pattern of APP in human patient specimens. To interrogate the functional role of APP in cell growth and apoptosis, the effect of APP knockdown in MDA-MB-231 cells were analyzed. Specifically, multiple signal transduction pathways and functional alterations linked to cell survival and motility were examined in *in vivo* animal model as well as *in vitro* cell culture with the manipulation of APP expression.

**Results:**

We found that the expression of APP is increased in mouse and human breast cancer cell lines, especially in the cell line possessing higher metastatic potential. Moreover, the analysis of human breast cancer tissues revealed a significant correlation between the level of APP and tumor development. Knockdown of APP (APP-kd) in breast cancer cells caused the retardation of cell growth *in vitro* and *in vivo,* with both the induction of p27^kip1^ and caspase-3-mediated apoptosis. APP-kd cells also had higher sensitivity to treatment of chemotherapeutic agents, TRAIL and 5-FU. Such anti-tumorigenic effects shown in the APP-kd cells partially came from reduced pro-survival AKT activation in response to IGF-1, leading to activation of key signaling regulators for cell growth, survival, and pro-apoptotic events such as GSK3-β and FOXO1. Notably, knock-down of APP in metastatic breast cancer cells limited cell migration and invasion ability upon stimulation of IGF-1.

**Conclusion:**

The present data strongly suggest that the increase of APP expression is causally linked to tumorigenicity as well as invasion of aggressive breast cancer and, therefore, the targeting of APP may be an effective therapy for breast cancer.

## Background

Amyloid-β precursor protein (APP) is a highly conserved single transmembrane protein with a receptor-like structure and has been linked with Alzheimer disease [1, 2] while its normal physiological function is unclear. Several APP isoforms derived from alternative splicing processes have been reported and diverse products including soluble APP (sAPP) or abnormal amyloid-β peptide through α-, β-, or γ-secretase-mediated cleavage(s) are post-translationally generated [3, 4]. APP is ubiquitously expressed in a broad spectrum of cell types including non-neuronal cells, while the nature of APP has been mainly studied in neuronal cells due to its pathological significance in Alzheimer disease. Several pathophysiological functions of APP have been proposed such as its potential role in cell growth and cell adherence [5–7]. It has been demonstrated that APP is engaged in neuronal growth cone adhesion and plays a role as an independently operating cell adhesion molecule for binding to extracellular matrices such as laminin [6]. Specifically, it has been reported that APP is linked to proliferation of thyroid epithelial cells and epidermal basal cell proliferation [8–11] and, interestingly, the increased expression of APP in several types of cancers including pancreatic, lung, colon and breast cancer has been reported [10–15]. These studies suggested that APP has growth-promoting effect as an autocrine growth factor while the underlying mechanism in the regulation of cellular signaling and gene expression has not been fully explored. The potential role of APP in cancer cell motility is also supported by studies which show APP plays a role in migration of neuronal precursor cells and neurite outgrowth [16, 17].

In this study, we explored the pathological role of APP in malignancy of breast cancer and its potential molecular mechanism related with cell proliferation and metastasis. Breast cancer is the most common cancer diagnosed among women worldwide [18] and metastatic breast cancer is significantly correlated with poor prognosis and a main cause of death while the underlying molecular pathogenic mechanism still remains to be delineated. We found that the expression level of APP is mechanistically linked with tumorigenicity and malignancy of breast cancer. APP knockdown (APP-kd) in breast cancer cells reduced cell growth via p27^kip1^ induction, promoting apoptosis, increasing sensitivity to therapeutic treatments, and delayed cell migration and invasion ability upon stimulation. These results suggest that targeting APP may effectively suppress the growth and invasion of malignant breast cancer cells.

## Methods

### Cell culture and reagents

MDA-MB-231 cells were grown in DMEM, and 67NR, 4T07, and 4T1 breast cancer cell lines were grown in RPMI supplemented with 10% (vol/vol) FBS, penicillin (100 units/ml), and streptomycin (100 μg/ml; Invitrogen, Rockville, MD). The four human breast cancer cell lines MCF10A1 (M-I), MCF10AT1k.cl2 (M-II), MCF10CA1h (M-III), and MCF10CA1a.cl1 (M-IV) were obtained from Dr. Anita Roberts (NCI/NIH, Bethesda, MD). M-I, M-II, M-III, and M-IV cells were grown in DMEM/F12 (Invitrogen, Carlsbad, CA) containing 5% horse serum (Invitrogen) at 37°C with 5% CO_2_. M-I and M-II cells were supplemented additionally with 10 μg/ml insulin (Sigma, St. Louis, MO), 20 ng/ml epidermal growth factor (Sigma), 0.5 μg/ml hydrocortisone (Sigma), and 100 ng/ml cholera toxin (Sigma). Antibodies specific for APP (22C11) were purchased from EMD Millipore; APP (4G8) from Covance. Specific antibodies for p27(C-19) and p21 (F-5) were from Santacruz and anti-β-actin (AC-15) was from Sigma. Antibodies purchased from Cell Signaling were AKT (#9772), pAKT Thr308 (#4056), pAKT Ser473 (#9271), pFOXO1 Thr24 (#9464), pGSK3 Ser9 (#9336), pp65 Ser536 (#3033), pERK1/2 (#9101), β-Catenin (#9562), PARP (#9542), and cleaved Caspase-3 (#9661). Anti-survivin antibody (AB8228) was purchased from Abcam. The anti-CD44 antibody (#15675-1-AP) was from Proteintech group and anti-GSK3b (KAP-ST002E) antibody was from Stressgen.

### Knockdown of human APP using lentiviral infection system

Knockdown of human endogenous APP gene expression was carried out using the lentivirus shRNA expression system and experimental method as previously described [19]. The target sequence of human APP (shAPP-5: 5’-CCCTGTTCATTGTAAGCACTT, shAPP-7: 5’-GCAGACACAGACTATGCAGAT) or control luciferase was used. In order to produce viral particles, the shRNA constructs and virus packaging plasmids were transfected into fresh 293T cells and then harvested the viral supernatant and filtered through 0.45 μm syringe filter prior to infection. Target cells were infected with virus by spinning at 2000 rpm for 30 min. Semi-quantitative RT-PCR and immunoblotting were carried out to measure knock-down efficiency.

### Western blotting and RT-PCR

The cells were harvested and lysed in RIPA buffer. Equal amounts of protein were loaded and separated in SDS-PAGE gel and then transferred to PVDF membrane. The blot was incubated in blocking solution (5% milk/TBST) and then incubated with primary antibody followed by incubation with secondary HRP conjugated antibody for 1 or 2 hours. The blot was washed 3 times for 5 minutes with TBST between the incubations. Eventually, the change of target protein expression was detected by conducting reaction with Chemiluminescent Substrate (Thermo Scientific), exposing, and developing the film. RT-PCR for measuring the level of APP mRNA expression was performed with the primers specific to human APP [20].

### Detection of apoptotic cell population

MDA-MB-231 cells (5×10^4^) freshly infected with shLuc, shAPP-5, or shAPP-7 lentiviral particles were immediately seeded in 6-well plates. In order to detect early apoptotic events, we employed Annexin V staining method (eBioscience) which can detect phosphatidylserine on the outer plasma membrane upon initiation of apoptosis. Cell viability staining was carried out using propidium iodide (PI) to identify early-stage apoptotic cells. The FACS analysis was immediately followed after staining the cells.

### Cell growth assay

The control and APP-kd of MDA-MB-231cells (2×10^3^) were seeded in 6-well plate in triplicate and maintained in normal growth medium. The sub-confluently growing cells were counted using coulter counter (Beckman) at day 2 and 4.

### Wound-healing assay and cell invasion assay

To compare the cell motility, the MDA-MB-231 control (shluc) or APP knockdown (shAPP-7) MDA-MB-231 cells were examined in wound healing assay. The confluently grown cells were wounded with 200 μl tips and followed by either no treatment or treated with IGF-1 (25 ng/ml) for 18 hours in 0.1% serum containing medium. Subsequently, cells were fixed with 2% paraformaldehyde and then stained with rapid 3 step staining set (Richard-Allen Scientific) for clear visualization of migrated cells. The initial wounded edges were marked with dotted lines. Representative results from at least three independent experiments are shown. Cell invasion assays were performed by seeding cells in Boyden chamber (BD Bioscience) coated with matrigel in serum-free medium with or without IGF-1 (50 ng/ml) in the bottom of each wells for 18 hours. The migrated cells were visualized by staining and photographing under the microscope.

### Xenograft mouse model

The breast cancer cells were seeded freshly prior to injection. The control and shAPP MDA-MB-231 (1×10^6^) cells were prepared in the solution (1:1) of PBS and growth factor-reduced matrigel and followed by injection into athymic nude mice subcutaneously. Primary tumor outgrowth was monitored every 4 days by taking measurements of the tumor length (*L*) and width (*W*). Tumor volume was calculated as π*LW*^2^/6 [21]. The mice were maintained up to 6 weeks and sacrificed for tumor excision. The tumor growth was compared to the counterpart and imaged. All animal housing and procedures were performed in compliance with guidelines established by the Institutional Animal Care and Use Committee at Case Western Reserve University.

### Immunohistochemistry

The breast cancer tissue array was purchased from US Biomax (Cat# BRC961). For immunohistochemistry for the APP detection, the tissue microarrays were hydrated through two changes of xylene and descending ethanol solutions for 10 min each, followed by a 30 min submersion in 3% H_2_O_2_ and finally Tris-buffered saline (TBS). The slides were incubated in 10% normal goat serum (NGS) in TBS for 30 min and the primary antibody was applied overnight. A monoclonal antibody specific to APP, 22C11 (recognizing the N-terminal domain of full length amyloid-β precursor protein; EMD Millipore, 1:250), was applied to the microarrays and then the peroxidase-anti-peroxidase technique was employed and developed with 3′-3’-diaminobenzidine (Dako).

### Statistical analysis

Data are presented as means ± standard deviation. Differences between the experimental groups were compared with Student’s paired two tailed t-test. A p-value less than 0.05 was considered statistically significant.

## Results

### The level of APP expression is linked to malignancy of breast cancer cells

**Figure 1:**
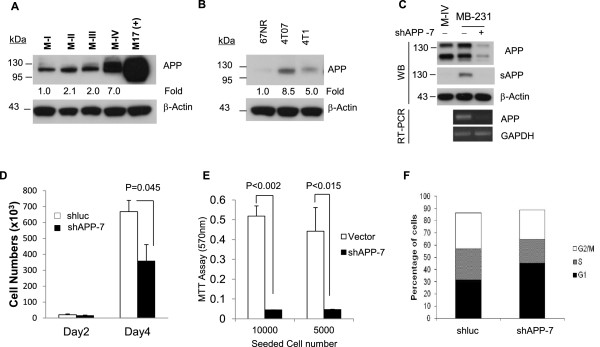
**The elevated expression of APP engaged in breast cancer cell proliferation. (A)** APP expression is detected by 22C11 mouse monoclonal anti-APP antibody in human breast cancer cell lines and correlates with increasing malignancy. (+); a positive control of APP protein overexpressed in neuronal cells. **(B)** The expression of APP is compared in mouse breast cancer cells with increasing metastatic potential. **(C)** APP protein expression was present at a similar level in both M-IV and MDA-MB-231. Knock down of APP expression was verified in RT-PCR following lentiviral infection encoding shAPP in MDA-MB-231. APP knockdown resulted in decreased expression of APP and soluble APP. The equal volume of conditioned media was condensed by using Centricon and analyzed in Western blot. For the loading control, β-actin was uesd. **(D)** Cells (2x10^3^) were seeded in 6-well plate and cell numbers counted using coulter counter at day 2 and 4. **(E)** MDA-MB-231 cells were seeded at two different numbers and the cell growth was compared by MTT assay. **(F)** MDA-MB-231 cells fixed and stained with propidium iodide (PI) were subjected to cell cycle analysis by FACS.

In order to investigate the correlation between APP expression and malignancy of breast cancer, the expression level of APP was examined in a series of human and mouse breast cancers with increasing malignancy. The four human breast cancer cell lines MCF10A1 (M-I), MCF10AT1k.cl2 (M-II), MCF10CA1h (M-III), and MCF10CA1a.cl1 (M-IV) were used in which M-I cells are spontaneously immortalized from normal breast epithelial cells whereas M-II, M-III, and M-IV cells are derived from M-I cells transformed with Ha-Ras oncogene [22, 23]. M-III cells are a well-differentiated tumor derived from M-II xenografts while M-IV cells are a poorly differentiated metastatic tumor derived from xenografts of M-II cells. In our analysis, the total APP expression of both mature (upper band) and immature (lower band) forms was significantly elevated approximately 2 to 7-fold in MCF10A (M-II, -III, and -IV) cells compared to M-I cells (Figure 1A). This positive correlation between APP expression and malignancy was further confirmed in mouse breast cancer cells; 67NR, 4T07, and 4T1 cells which are derived from the same primary tumor [24]. 67NR cells, which can form primary tumors without metastatic ability, showed negligible APP expression whereas highly tumorigenic 4T07 and metastatic 4T1 cells express APP up to 8-fold (Figure 1B). These results suggest that APP is functionally linked to the aggressiveness in breast tumor cells and contribute to maintaining their malignancy such as tumorigenic and metastatic ability.

### Reduction of the expression of APP prevents cell growth in MDA-MB-231 cells

We investigated the pathophysiological function of APP by knocking it down using the shRNA targeting APP in MDA-MB-231 malignant human breast cancer cells (Figure 1A). Both mRNA and protein expression of APP were markedly reduced in APP-kd cells compared to control cells (Figure 1C). APP protein expression of MDA-MB-231 was comparable to that of M-IV cells while MDA-MB-231, but not M-IV cells, showed fair amount of soluble APP secretion that is known to enhance cell growth and survival [25, 26]. Next, we examined cell proliferation in normal growth medium with 10% FBS in the control (shluc) and APP-kd (shAPP) cells. Consistent with our hypothesis, reduction of APP expression significantly affected cell proliferation and viability (Figure 1D,E). To confirm the effect of APP on cell growth further, we performed FACS analysis to determine cell cycle phase. The cell cycle analysis showed that APP-kd cells were arrested largely in G1 phase (45.2%) compared to control (31.4%), but low percentage of APP-kd cells (19.4%) was in S phase as compared to that of control cells (25.5%) (Figure 1F). Retarded cell growth and G1 arrest of APP-kd cells suggest that APP is likely engaged in expression of cell cycle inhibitors working on G1 phase such as p27^kip1^ and p21^cip1^ [27, 28].

### APP enhances cell proliferation via regulation of p27^kip1^

**Figure 2:**
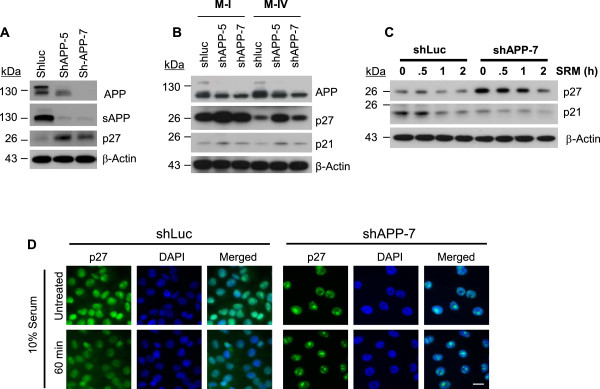
**APP involved in the induction of cell cycle inhibitor p27** ^**kip1**^ **in breast cancer cells. (A)** Knock-down of APP in MDA-MB-231 cells using two different shRNA constructs of APP (shAPP-5 and shAPP-7) resulted in marked suppression of both cellular and soluble form of APP expression. The p27^kip1^ expression was elevated in shAPP-5 and shAPP-7 cells. **(B)** The p27^kip1^ and p21^cip1^ expression was evaluated in M-I and M-IV after introduction of shluc, shAPP-5, or shAPP-7. **(C)** The control and shAPP-7 cells were incubated in serum deprived medium for 3 hours and then released with 10% serum for the indicated time points. The cells were harvested and subjected to assessment of p27^kip1^ and p21^cip1^ expression. **(D)** The cells incubated in serum-free medium for 18 hours were treated with 10% serum for 60 minutes and then the images were acquired to show subcellular localization of p27^kip1^. The nuclear localized p27^kip1^ was confirmed by merging with DAPI images. The longer image acquisition was needed to detect p27^kip1^ in the control (shluc) cells due to the low expression of p27^kip1^. Scale bar = 20 μm.

To address whether APP regulates G1 phase cell cycle inhibitors, the control and APP-kd cells grown in normal growth medium were examined to compare p27^kip1^ and/or p21^cip1^ expression of APP-kd cells to control. In our analysis, the level of p27^kip1^ was dramatically induced in APP-kd cells compared to control (Figure 2A and 2B). However, p21^cip1^ expression was unchanged or slightly affected by APP knockdown in multiple cell lines (M-I, M-IV and MDA-MB-231) (Figure 2B and 2C) suggesting that APP regulates cell cycle by modulating p27^kip1^ specifically.

It has been established that p27^kip1^ has dual function as either a tumor suppressor or promoter because nuclear p27^Kip1^ works as an anti-proliferative protein, while cytoplasmic p27^kip1^ promotes cytoskeleton remodeling that is important for tumor cell motility and dissemination. In particular, subcellular location of p27^Kip1^ is significantly correlated with survival of breast cancer patients [29, 30]. In order to verify functional competency of p27^kip1^ as a cell cycle inhibitor, we analyzed cellular localization of p27^kip1^ with immunocytochemistry. A substantial amount of p27^kip1^ is still located in nuclear compartment of APP-kd cells even after one hour in serum-containing medium (Figure 2D). Conversely, in control cells, p27^kip1^ located in nuclei required much longer exposure time to be displayed owing to the substantial decrease of total protein with 10% serum stimulation, and potentially the redistribution of p27^kip1^ to cytoplasmic compartment. These results indicate that serum-sensitive signaling pathways regulating p27^kip1^ expression and cytoplasmic translocation were skewed by APP knockdown. These data also suggest that APP plays a crucial role for cell proliferation of malignant breast cancers by modulating the expression of cyclin-dependent kinase inhibitor, p27^kip1^.

### APP modulates breast cancer cell survival

**Figure 3:**
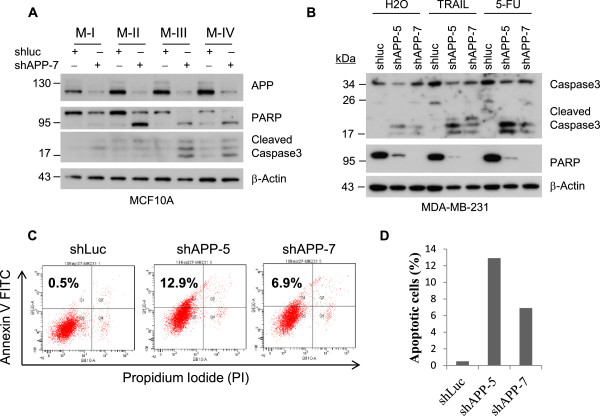
**Reduction of APP expression is associated with the apoptotic induction in breast cancer cells. (A)** A series of MCF-10A cells were infected with lentivirus encoding control (shluc) or APP shRNA (shAPP-7) and then tested for APP expression by immunoblotting. Under this condition, alteration of apoptotic indicators such as cleaved PARP and cleaved Caspase-3 were compared. **(B)** MDA-MB-231 cells were infected with lentivirus encoding shluc, shAPP-5, or shAPP-7. Each cell line was treated with TRAIL (10 ng/ml) or 5-FU (200 μM) for 24 hours. **(C, D)** The on-going early apoptotic events were compared by staining for extracellular Annexin V and cell viability with propidium iodide (PI). The apoptotic cell populations with Annexin V high and PI low were indicated as percentage.

The reduction of breast tumor growth may result not only from blocking cell cycle progression but also the induction of programmed cell death. Thus, we examined if knockdown of APP expression induces cell death in MCF10A and MDA-MB-231 cell lines. Knocking down of APP in M-II cells significantly induced apoptotic markers such as cleavage product of PARP and cleaved caspase-3 in contrast to the normal immortalized M-I cells which did not sensitively induce such apoptotic markers. Moreover, M-III and M-IV showed such apoptotic markers to a much greater extent (Figure 3A), suggesting that the cell survival of advanced breast cancer cells is more dependent on APP expression than non-malignant breast epithelial cells (M-I). Next, we assessed the induction of apoptotic markers in MDA-MB-231 and the sensitivity to therapeutic agents such as recombinant tumor necrosis factor (TNF)-related apoptosis-inducing ligand (TRAIL), or 5-Fluorouracil (5-FU). TRAIL has been tested as a potential therapeutic agent for various types of cancer in clinical trials [31], and 5-FU is a conventional chemotherapeutic agent that is commonly used for cancer therapy [32]. The cleaved capase-3 and PARP were augmented in MDA-MB-231 APP-kd cells (shAPP-5 or shAPP-7) (Figure 3B) which were consistent with the results from M-III and M-IV cells (Figure 3A). The induction of apoptosis by knockdown of APP was also confirmed by FACS analysis with staining for Annexin V and propidium iodide (PI). The apoptotic cell populations with Annexin V-high and PI-low were obviously increased in APP-kd cells showing about 25-fold (shAPP-5) and 14-fold (shAPP-7) induction as compared to control (Figure 3C and 3D). These results clearly indicate that APP expression on breast cancer cells is closely interelated with cell survival.

### APP affects cell growth in 3D culture and in xenografted mouse model

**Figure 4:**
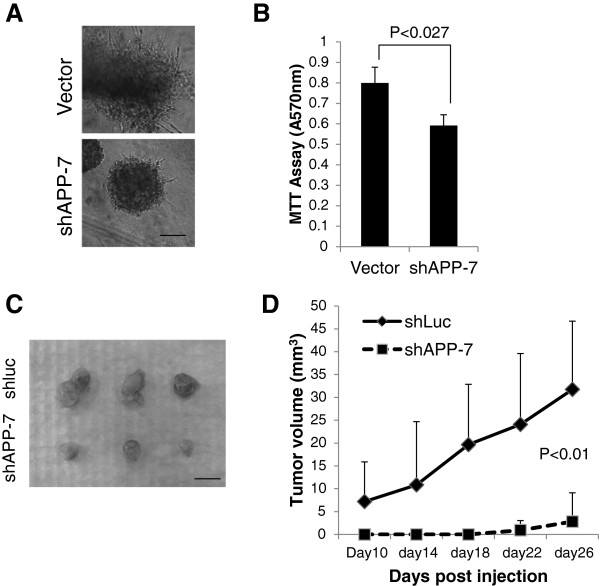
**APP modulates breast cancer cell growth in 3D culture and in xenografted model.** MDA-MB-231 cells were subjected to 3D Matrigel on-top assay. The cells were seeded (2x10^4^/well) in 48-well plate coated with Matrigel in triplicate and then cultured for 7 days with medium change in every two days. The morphology of growing cells were obtained **(A)** and followed by MTT assay **(B)**. **(C)** The control and shAPP-7 MDA-MB-231 (2x10^6^) cells were injected into nude mice s.c. (n = 6) and allowed to grow for 6 weeks. The grown tumors were excised and the grown tumor size compared. (Scale bar = 1cm) **(D)** The independent xenograft study (2.5x10^5^ cells s.c injected; n = 5, respectively) revealed that shAPP-7 MDA-MB-231 cell growth rate was largely decreased as compared to control group (p < 0.01).

In order to solidify the finding of APP functions on cell growth, we employed three-dimensional (3D) cultures of breast cancer cells in reconstituted basement membrane (Matrigel, BD Bioscience). It is widely recognized that the 3D cultures offer many microenvironmental cues which reconstitute in vivo tumor cell behavior [33, 34]. The APP-kd MDA-MB-231 cells and its counterpart were cultured in 3D Matrigel up to 7 days. The control MDA-MB-231 cells showed higher tumor growth than APP-kd cells. Interestingly, control MDA-MB-231 cells showed stellate 3D phenotype whereas APP-kd cells displayed more round forms (Figure 4A and 4B). Since the characteristics of 3D morphology may represent functional and genetic alteration of cancer cells as shown in altered E-cadherin expression [35, 36], the 3D morphological change of APP-kd cells would result in behavioral and functional conversion. To confirm these *in vitro* findings further, we examined the effect of APP in the tumor xenograft mouse model. We injected the control or APP-kd MDA-MB-231 cells (2x10^6^) subcutaneously to nude mice and maintained the mice for 6 weeks. Consistent with the findings in cell culture models, APP-kd cells showed significantly reduced tumor forming ability *in vivo* compared to control (Figure 4C). As an independent experiment, we subcutaneously injected further reduced numbers (2.5×10^5^) of MDA-MB-231 cells (groups of control and APP-kd) and then measured tumor size over time. As a result of measurement up to 28-days post injection, there was a significant difference in tumor volume between control and APP-kd groups (Figure 4D). Tumor growth was negligible and difficult to measure in APP-kd group up to 22-days. These 3D culture and *in vivo* xenograft studies strongly support the role of APP in the promotion of breast cancer cell growth.

### APP is engaged in IGF1-induced AKT activation

**Figure 5:**
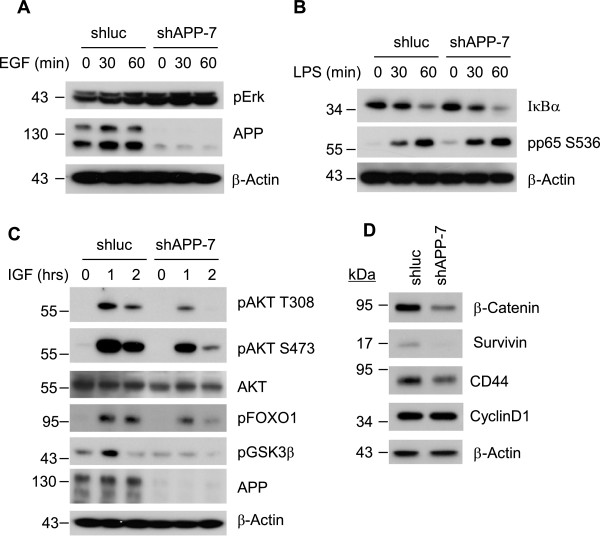
**APP significantly impacts IGF-1-mediated activation of AKT and its downstream effectors.** Both MDA-MB-231 control (shluc) and APP-kd (shAPP) cells were treated with EGF (50 ng/ml), LPS (100 ng/ml), or IGF-1 (100 ng/ml) as indicated. **(A)** EGF-mediated Erk activation was assessed in the APP knock-down cells post stimulation with EGF. **(B)** LPS-mediated activation of pro-inflammatory response in the APP knockdown cells was tested by demonstrating the level of IκBα expression and NF-κB activation (phosphorylated p65 at S536). **(C)** IGF-1-stimulated Akt activation and phosphorylation of Akt target proteins such as GSK3β (S9) and FOXO1 (T24) were examined. **(D)** APP affects the expression of β-Catenin, a target of GSK3β, and its downstream targets such as Survivin and CD44, but not Cyclin D1.

To understand the underlying mechanism of the effect of APP on breast cancer cells, we examined the signaling pathways potentially linked to p27^kip1^ and apoptotic induction in APP-kd cells. MDA-MB-231 cells are known to possess both K-Ras and B-Raf oncogenic mutations [37] which regulate ERK pathway. Thus, we examined the effect of APP-kd on ERK activation. After EGF treatment, APP knockdown failed to reduce ERK activation at both basal and EGF-stimulated conditions of MDA-MB-231 cells (Figure 5A). In addition, NF-κB activation, which is important for cell survival, was unaffected by APP knockdown, as indicated by similar level of I-kB degradation and p-p65 (Ser536) post LPS stimulation (Figure 5B), suggesting both pathways are not likely responsible either for p27^kip1^ or apoptotic induction in APP-kd cells. Next, we examined IGF-1/AKT signaling pathway in APP-kd cells since AKT/FOXO signaling axis have been identified as critical signaling intermediates for breast cancer survival, growth, and migration as well as therapeutic drug resistance [38, 39]. In the APP-kd cells, IGF-1-induced AKT phosphorylation at T308/S473 was evidently decreased over total Akt and, concurrently, AKT-mediated GSK3β phosphorylation at Ser 9 was reduced (Figure 5C). Knock down of APP also significantly reduced the phosphorylation of FOXO, a main substrate of AKT and a transcription factor that regulates cell cycle progression through induction of cell cycle inhibitors including p21^cip1^ and p27^kip1^. AKT is known to suppress FOXO family by inducing phosphorylation, nuclear export, and degradation which lead to subsequent p21^cip1^ and/or p27^kip1^ reduction [40]. AKT can also directly phosphorylate and regulate p27^kip1^ cytoplasmic redistribution [41]. As demonstrated in Figure 2, p27^kip1^ remained in the nucleus for a longer time in APP-kd cells after serum release. Thus, it is likely that mitigated AKT activation in APP-kd cells resulted in higher p27^kip1^ expression and prolonged retention in nucleus. Next, we examined the change of GSK3β downstream target proteins (Figure 5D). The expression of β-catenin and its downstream targets such as survivin and CD44, but not Cyclin D1 were affected by knockdown of APP likely through AKT-GSK3β axis. These findings indicate that elevated APP expression in breast cancer may promote cell growth and survival by the induction of AKT-FOXO and AKT- GSK3β signaling cascades.

### APP reduction reduces cell motility in MDA-MB-231 cells

**Figure 6:**
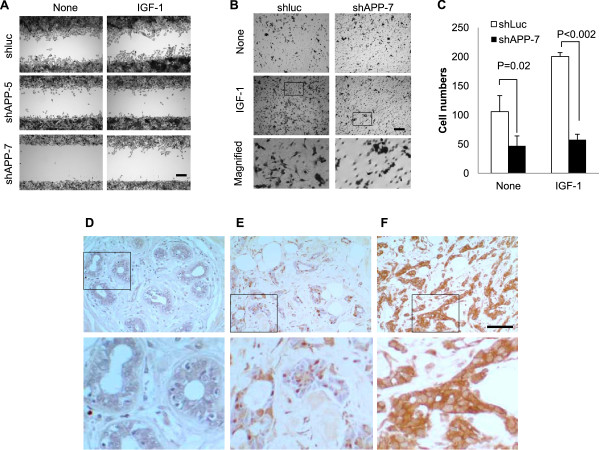
**APP promotes cell migration of MDA-MB-231 and its expression is elevated in invasive breast cancer of human tissues. (A)** The cell motility of APP knockdown (shAPP) MDA-MB-231 was examined in wound healing assay. Following the wounding, cells were untreated or treated with IGF-1 (25 ng/ml) for 18 hours in 0.1% serum containing medium. Cells were then fixed and stained for clear demonstration (scale bar = 200 μm). **(B)** The role of APP for cell migration was evaluated in Boyden chamber assay in serum-free medium with or without IGF-1 (50 ng/ml) for 18 hours. The rectangular area was further magnified for demonstration of different cell morphology. **(C)** The migrated cells in panel B were counted in three randomly selected areas. **(D)** No staining for APP (22C11) is present in this normal terminal duct lobular unit. **(E)** The well-differentiated grade 1 invasive ductal carcinoma shows weak staining for APP. **(F)** The poorly-differentiated grade 3 invasive ductal carcinoma shows strong staining for APP. Scale bar = 100 μm.

Since APP expression has been linked to cell migration [6, 16], we explored the role of APP in cell migration and invasion of MDA-MB-231. The confluent control (shLuc) and APP-kd (shAPP-5 or shAPP-7) cell cultures were wounded and allowed to migrate into the wounded area in low serum containing medium with or without IGF-1. APP-kd cells showed very limited cell migration into the wounded space compared to the control cells in the absence of any stimulation. Moreover, upon IGF-1 treatment, more substantial difference in cell migration was observed between control and APP-kd cells (Figure 6A). Next, we assessed the cell migration ability of APP-kd MDA-MB-231 cells in transwell chambers. As was observed in the wound healing assay, APP-kd cells exhibited limited migration ability with about 50% reduction in untreated cells and 75% reduction in IGF-1 treated cells (Figure 6B and 6C). Notably, MDA-MB-231 control cells treated with IGF-1 showed spindle-like mesenchymal cell morphology whereas APP-kd cells did not, suggesting the potential role of APP during cell invasion and metastasis through regulation of epithelial-mesenchymal transition (EMT). Taken together, our data indicate that APP is involved in the regulation of cell motility triggered by IGF-1 and APP might be an attractive therapeutic target to prevent cell invasion and metastasis.

### Increased expression of APP in human breast cancer tissues

In order to examine the clinical relevance of APP expression in breast cancer, a tissue microarray (TMA) containing various grades of breast cancer tissues and normal breast tissues was analyzed with an anti-APP antibody (22C11). In the normal breast tissues, there was minimal to no staining of the breast epithelium. However, the vast majority of the invasive breast carcinomas showed some degree of APP expression. In total, there were 40 invasive breast carcinomas that could be evaluated on the TMA sections stained with 22C11 antibody. No staining was observed in 3 (7.5%) of the cases. Weak staining was observed in 10 (25%) of the cases, moderate staining in 18 (45%), and strong staining in 9 (22.5%). Though the number of cases in this series is small, there was a trend seen where the higher grade tumors showed more intense staining than the lower grade tumors overall (Figure 6D-F). These results strongly support our hypothesis that elevated APP expression has close correlation with tumor cell growth and progression.

## Discussion

Our data strongly indicate the pathological role of APP in breast cancer. First, we demonstrated increased expression APP in breast cancer cells and its correlation with malignancy. Second, the inhibition of APP expression in breast cancer cells effectively prevents cell growth and motility *in vitro* and *in vivo* models. Third, we also demonstrated that APP is mechanistically linked to the AKT/FOXO and AKT/GSK3-β pathways which are known to modulate cell growth, survival, and invasion of breast cancer cells through the regulation of target genes including p27^kip1^ and survivin. Importantly, knocking down of APP expression resulted in retarded cell growth *in vitro* and *in vivo* xenografted mouse model. We found that the slower cell proliferation was, in part, caused by the upregulated cell cycle inhibitor p27^kip1^ expression in APP-kd cells. Thus, increased APP expression is inversely correlated with p27^kip1^ expression in malignant breast cancers. Since the reduced p27^kip1^ expression is correlated with tumor aggressiveness and poor patient survival [29], this finding suggests that APP plays a significant role in regulation of p27^kip1^ in a malignant human breast cancer. In addition, knockdown of APP in breast cancers augmented apoptotic markers and it is likely that advanced breast cancers (M-II, M-III, and M-IV) with knockdown of APP are more prone to enter into apoptosis. Similarly, in addition to the result of MCF-10A cells, APP knockdown in MDA-MB-231 promotes sensitivity to therapeutic treatments of TRAIL or 5-FU, implying that targeting APP in malignant breast cancers may promote the sensitivity to therapeutic drugs. Since homozygous APP-deficient mice are viable and normal in development [42], it seems that normal breast epithelial cell growth is not affected by knockdown of APP expression. However, advanced breast cancers may struggle to survive in the absence of APP, presumably because they have evolved to survive better, at least in part, in an APP-dependent manner. After the submission of this manuscript, Goodarzi et al. [43] published an article demonstrating the biological effect of APP in the regulation of breast cancer progression. Their results suggest that APP might suppress aggressiveness of breast cancer cells. While those results are not overlapped with the phenotype of our APP knockdown experiments, both reports strongly suggest the pathological role of APP in breast cancer pathogenesis. The discrepancy between two studies might be explained by different cellular conditions used in the studies. While they examined the role of APP under the condition of TARBP2 knockdown, our study examined a direct function of APP in the parental MDA-MB-231 cells without any other combinatorial genetic modifications. These results strongly suggest that the pathological role of APP in breast cancer pathogenesis works diversely upon the cellular context and this needs to be addressed in the future study.

Our data also suggest that APP is involved in IGF-1/AKT signaling pathways, which are key regulatory pathways for cell growth and survival of breast cancer. APP-kd cells displayed mitigated AKT activation which leads to decreased inhibitory phosphorylation of GSK3β (Ser9) and FOXO1 (T24). GSK3β is known to suppress β-catenin-dependent oncogenic signaling pathway by phosphorylating β-Catenin [44, 45]. Activation of β-catenin is reported in subgroup of triple negative breast cancers (i.e., aggressive breast cancers possessing lack of estrogen receptor, progesterone receptor, and Her2 receptor expression) and is associated with poor clinical outcomes [44]. On the other hand, FOXO family including FOXO1 can induce cell cycle inhibitors (e.g., p27^kip1^, p21^cip1^) and pro-apoptotic molecules (e.g., BIM, BNIP3, FASL, TRAIL, and survivin) [46]. The anti-apoptotic protein, survivin, is a family member of inhibitors of apoptosis (IAP) which embodies diverse cellular function, encompassing mitosis, metabolism, and survival by promoting adaptation to stresses [47]. As such, FOXO-survivin and β-catenin-survivin regulatory pathways are considered to play an essential role for the expression of survivin in breast cancer [38, 44]. Thus, our results strongly suggest that APP-mediated regulation of AKT/FOXO and AKT/GSK3β pathways are playing a significant role for breast cancer development. Supporting this hypothesis, a previous study demonstrated that sAPPα stimulates AKT/GSK3β pathway in neuronal cells and consequently resulted in its neuroprotective effect [48].

Interestingly, APP is also known to promote cell migration in neuronal progenitor cells [16] and engage in neuronal growth cone adhesion where it plays a role as an independently operating cell adhesion molecule for binding to extracellular matrices such as laminin [6]. Acquiring cell motility is a key aspect enabling cancer cells to invade into adjacent tissue and disseminate into the secondary organs. We therefore examined the cell motility and invasion ability of MDA-MB-231 after knocking down of APP expression. Upon stimulation with IGF-1 that promotes cell migration and cancer metastasis, APP-kd cells migrate slowly in response to IGF-1 partly due to limited activation of AKT. It is well known that AKT plays an important role in the process of EMT via repression of E-cadhrin [49]. In addition, β-catenin is also closely engaged in EMT and cell migration [50, 51]. Our findings that APP is functionally linked with AKT activation and GSK-3β/β-catenin pathways warrant the future study that elevated APP in malignant breast cancers is associated with dissemination of breast cancer into other target organs by promoting EMT process.

## Conclusions

In summary, we found that the expression of APP is increased both in mouse and human malignant breast cancer cell lines and similarly in human breast cancer tissues. The APP expression is important to regulate cell growth, apoptosis, and motility of breast cancer, possibly through engagement of AKT-mediated signaling pathways. Overall, our findings provide substantial groundwork for the pathophysiological function of APP and its underlying mechanism that promotes breast cancer malignancy.

## Abbreviations

APP: Amyloid-β precursor protein
TMA: Tissue microArray
IGF-1: Insulin-like growth factor-1
DAPI: Diamidino-2-phenylindole
TRAIL: Tumor necrosis factor (TNF)-related apoptosis-inducing ligand
5-FU: 5-Fluorouracil.
